# Potential Antioxidant and Anti-Inflammatory Effects of *Lonicera japonica* and *Citri Reticulatae Pericarpium* Polyphenolic Extract (LCPE)

**DOI:** 10.3390/antiox12081582

**Published:** 2023-08-08

**Authors:** Se Hyo Jeong, Min Yeong Park, Pritam Bhagwan Bhosale, Abuyaseer Abusaliya, Chung Kil Won, Kwang Il Park, Eunhye Kim, Jeong Doo Heo, Hyun Wook Kim, Meejung Ahn, Je Kyung Seong, Hun Hwan Kim, Gon Sup Kim

**Affiliations:** 1 Research Institute of Life Science and College of Veterinary Medicine, Gyeongsang National University, 501 Jinju-daero, Jinju 52828, Republic of Korea; tpgy123@gmail.com (S.H.J.); lilie17@daum.net (M.Y.P.); shelake.pritam@gmail.com (P.B.B.); yaseerbiotech21@gmail.com (A.A.);; 2 Biological Resources Research Group, Gyeongnam Department of Environment Toxicology and Chemistry, Korea Institute of Toxicology, 17 Jegok-gil, Jinju 52834, Republic of Korea; 3 Division of Animal Bioscience & Intergrated Biotechnology, Jinju 52725, Republic of Korea; 4 Department of Animal Science, College of Life Science, Sangji University, Wonju 26339, Republic of Korea; 5 Laboratory of Developmental Biology and Genomics, BK21 PLUS Program for Creative Veterinary Science Research, Research Institute for Veterinary Science, College of Veterinary Medicine, Seoul National University, Seoul 08826, Republic of Korea

**Keywords:** HPLC-MS/MS, LCPE, antioxidant, anti-inflammation, keratinocytes

## Abstract

Dermatitis is an inflammatory condition of the outer layer of the skin that causes itching, blisters, redness, swelling, and often exudation, scabs, and peeling. Among them, purulent inflammation is a symptom that often occurs on the skin and appears in the form of boils and acne. Various studies are being conducted to treat these inflammatory diseases. Accordingly, *Lonicera japonica* and *Citri Reticulatae Pericarpium* Polyphenolic Extract (LCPE), which uses herbal preparations such as *Lonicera japonica*, *Citri Reticulatae Pericarpium*, and *Glycyrrhiza uralensis*, has been used to suppress inflammation since ancient times, and its anti-inflammatory effect can be observed in skin keratinocytes after inducing inflammation. In this study, the major polyphenolic compounds in LCPE were quantitatively determined by analyzing the data through peak values using high-performance chromatography (HPLC-MS/MS) coupled with mass spectrometry. Additionally, bioactive compounds targeting 2,2-diphenyl-1-picrylhydrazyl (DPPH) were analyzed by ultrafiltration integrated with LC. Several compounds with the most significant effects were selected (chlorogenic acid, narirutin, and isorhamnetin). Skin keratinocytes induced by lipopolysaccharide (LPS) were treated with LCPE to show its anti-inflammatory effects. After LCPE treatment, inflammation-mediating cytokines such as cyclooxygenase-2 (COX-2) and inducible nitric oxide synthase (iNOS) were decreased. In addition, nuclear factor kappa (NF-кB) and mitogen-activated protein kinase (MAPK) were inhibited in important pathways related to inflammation. Lastly, molecular modeling was performed to determine binding scores with inflammation-related proteins using molecular docking for the selected compounds. According to these results, LCPE is effective in treating keratinocytes induced by LPS and reducing inflammation and has potential antioxidant effects, and the polyphenol components have been identified.

## 1. Introduction

Since the skin is an organ that plays an important role in protecting against various types of external damage, the inflammatory response in the skin plays a particularly important role in defense and homeostasis [1]. Dermatitis largely includes atopic, contact, and seborrheic types. Atopic dermatitis is an inflammatory disease caused by genetic effects on proteins and immune cells constituting the skin. Contact dermatitis is itchy because of contact with external substances and is characterized by inflammation of the skin. Seborrheic dermatitis is an inflammatory skin disease that occurs in areas rich in sebaceous glands [2,3].

*Lonicera japonica* and *Citri Reticulatae Pericarpium* Polyphenolic Extract (LCPE) is an extract containing herbal ingredients such as *Lonicera japonica*, *Citri Reticulatae Pericarpium*, and *Glycyrrhiza uralensis*, in addition to *Taraxacum* and *Forsythia suspensa*. *Lonicera japonica* contains various active substances, such as saponins, flavonoids, tannins, and alkaloids, and has excellent anti-inflammatory, antiviral, antitumor, and antioxidant effects [4]. The citrus peel of *Citri Reticulatae Pericarpium* (Chenpi) contains many physiologically active substances, such as flavonoids, which are especially abundant in tangerines. It reduces stress and is effective for skin diseases such as purulent acne, eczema, and itching [5]. *Glycyrrhiza uralensis* also contains saponin or flavonoids; it is effective in anticancer, immune regulation, and anti-inflammatory treatment and is an excellent medicine [6].

Prostaglandins (PGs) and free radicals, substances created by metabolism, are the principal causes of oxidative and inflammatory stress [7]. Antioxidant action is mediated by a variety of mechanisms, the main one of which is the suppression of reactive oxygen species (ROS) generation. These include the generation of reactive oxygen species (ROS), ROS removal, the activation of antioxidant defenses, or the suppression of defense-related enzymes [8]. Inflammation is a kind of defense mechanism caused by various immune responses and the activation of vascular cells when tissue damage or infection occurs [9]. This antioxidant effect is strongly related to the suppression of inflammatory responses by preventing tissue damage. In particular, natural-compound-based antioxidants are considered promising therapeutic agents for reducing oxidative stress by suppressing free radical production [10]. Lipopolysaccharide (LPS) is a substance that forms the cell membrane of Gram-negative bacteria. It acts as an intracellular toxin and stimulates macrophages to activate NF-кB to induce inflammation [11]. When inflammation is induced by LPS, inflammatory mediators such as nitric oxide (NO) and prostaglandin E2 (PGE2) are activated [12]. In addition, inflammatory cytokines such as cyclooxygenase-2 (COX2) and inducible nitric oxide synthase (iNOS) are also upregulated through the phosphorylation of IкBα and P65 in the NF-кB pathway and the phosphorylation of JNK, ERK1/2, and P38 in the MAPK pathway [13].

Since molecular docking accurately predicts the shapes of protein–ligand binding sites, it is frequently utilized for structure-based drug design and the prediction of functional sites on protein molecules’ surfaces [14]. It is important that the scores displayed throughout the docking process are not evaluated by determining the precise binding affinity. As a result, the step of visually verifying structural binding is necessary to confirm the molecular docking result [15].

In this study, first, the main polyphenol components of the complex extract were identified, and polyphenol compounds contained in LCPE were identified through HPLC-MS/MS for further study. The excellent antioxidant effect of the compound was confirmed through the combination of HPLC with DPPH. Afterward, to confirm whether this LCPE composite extract has an anti-inflammatory effect using HaCaT cells, a skin keratinocyte cell line, inflammation was induced by LPS in order to confirm its potential as an excellent dermatitis remedy candidate.

## 2. Materials and Methods

### 2.1. Plant Materials

For the experiments, plants from the hilly region of Geochang, Gyeongsangnam-do, Korea, including *Lonicera japonica* and *Glycyrrhiza uralensis*, were employed. *Citri Reticulatae Pericarpium* was collected from abandoned farms on Jeju Island. The harvested plants were rinsed in water and then chopped and dried for 72 h in a 56 °C dry oven. After that, they were kept at −20 °C in sealed polyethylene bags containing silica gel until usage.

### 2.2. Reagents, Chemicals, and Standards

The DPPH (2,2-Diphenyl-1-picrylhdrazyl) reagent and standard compounds were purchased from Sigma-Aldrich Corp (St. Louis, MO, USA, cas no. 1898-66-4). A centrifugal ultrafiltration filter (YM-30) with a capacity of 30 kDa (Millipore Co., Ltd., Darmstadt, Germany) was purchased. All other chemicals and solvents utilized were of analytical grade (Duksan Pure Chemical Co., Ltd., Ansan, Republic of Korea). Fetal bovine serum (FBS), phosphate-buffered saline (PBS), Dulbecco’s modified Eagle’s medium (DMEM), and penicillin/streptomycin (P/S) antibiotics were acquired from Gibco (BRL Life Technologies, Grand Island, NY, USA). Antibodies against COX-2 (cat. no. 12282S), iNOS (cat. no. 13120S), p65 (cat. no. 8242S), phosphorylated p65 (p-p65) (cat. no. 3033S), IкBα (cat. no. 4812S), phosphorylated IкBα (p-IкBα) (cat. no. 2859S), JNK (Jun N-terminal kinase) (cat. no. 9258S), phosphorylated JNK (p-JNK) (cat. no. 4671S), ERK (extracellular-signal-regulated kinase) (cat. no. 4695S), phosphorylated ERK (p-ERK) (cat. no. 4370S), p38 (cat. no. 8690S), phosphorylated p38 (p-p38) (cat. no. 9216S), and β-actin (cat. no. 3700S) were purchased from Cell Signaling Technology (Danvers, MA, USA). Horseradish peroxidase-conjugated secondary antibodies to anti-rabbit (cat. no. A120-101P) and anti-mouse (cat. no. A90-116P) were purchased from Bethyl Laboratories, Inc. (Montgomery, AL, USA).

### 2.3. Extraction Process of LCPE and Purification of Polyphenol Components

Polyphenols were isolated from plants using a modified technique with LCPE [16]. Herbs such as dried lonicera flower 15 g, dandelion 10 g, citrus peel 10 g, forsythia fruit 10 g, and licorice 5 g were extracted with 4 L of 70% methanol for 4 days. The mixture was filtered through Whatman qualitative No. 6 filter paper. A rotary evaporator (N-1110, Eyela, Tokyo, Japan) operating at 100 revolutions per minute was used to concentrate the mixture to 500 mL at reduced pressure and 45 °C. The concentrate was washed three times with 500 mL of hexane to remove fatty particles. The remaining filtrate was extracted three times with 250 mL of ethyl acetate. The residue was first dehydrated with MgSO_4_ and then eluted with silica gel solvent (40 cm × 2.5 cm) and ethyl acetate to remove highly polar substances. Under lower pressure, the solvent was condensed to produce a mixed polyphenol powder and stored at −70 °C (1.4 g, 2.8% of dried raw plant materials).

### 2.4. HPLC and LC-MS/MS

HPLC and LC-MS/MS were carried out using a 3200 QTrap Tandem Mass System (Sciex LLC) and a 1260 Series HPLC System (Agilent Technologies, Inc., Santa Clara, CA, USA) in positive-ion mode with the spray voltage set to −4.5 kV. The gradient system was set at a flow rate of 0.5 mL/min, and the solvents were DW and acetonitrile with 0.1% formic acid. The analysis was performed using an analytical Prontosil C18 column from Phenomenex Co., Ltd., in Torrance, CA, USA (length: 250 mm; inner diameter: 4.6 mm; particle size: 5 µm). The solvent conditions in the mobile phase were 0–10 min at 10–15% Acetonitrile (ACN), 10–20 min at 20% ACN, 20–30 min at 25%, 30–40 min at 40%, 40~50 min at 70%, 50–60 min at 95%, and 60–70 min at 95%. The analysis was conducted at a wavelength of 284 nm and 35 °C. Peak areas acquired from UV and reference materials were used to calculate the amounts of polyphenolic chemicals present.

### 2.5. DPPH-Binding HPLC Analysis for Measuring Main Antioxidant Activity of Polyphenolic Compounds

Polyphenolic compounds (1250 µg/mL) and 0.2 mg/mL DPPH reagent were mixed in a ratio of 1:1 (*v*/*v*) and reacted at room temperature for 15 min. The mixture was filtered using a 0.45 m filter before being HPLC-analyzed, and methanol was used as a control in place of the DPPH reagent. The content of the substance that reacted with DPPH could be determined by examining the chromatographic peak values and standard curve values of the samples and controls that underwent the DPPH reaction. Through this analysis, the main antioxidant components among LCPE phenolic compounds were identified.

### 2.6. Measurement of Anti-Inflammatory Effects

#### 2.6.1. Cell Culture and Viability Assay

The skin keratinocyte HaCaT cells were obtained from the American Type Culture Collection (ATCC) and were cultured in complete DMEM containing 10% FBS and supplemented with 100 U/mL penicillin and 100 µg/mL streptomycin (P/S). The cells were incubated at 37 °C in a humidified atmosphere containing 5% CO_2_.

HaCaT cells were seeded at a density of 1 × 10^4^ cells per well in 96-well plates for 12 h. The cells were then treated with LCPE at concentrations of 0, 0.1, 0.25, 0.5, 0.75, 1, 1.25, 2, 2.5, 5, 7.5, and 10 µg/mL for 24 h, either with or without 1 µg/mL LPS (Sigma-Aldrich, Merck KGaA, Burlington, MA, USA). Each well was treated with 10 µL of MTT solution (5 mg/mL) before the cells were cultured for 4 h at 37 °C. DMSO was used to dissolve the crystals of formazan that were insoluble. Lastly, each sample was analyzed in triplicate, and the optical density (OD) value of each well was read at 450 nm using a microplate reader (BioTek, Winooski, VT, USA).

#### 2.6.2. Western Blot Analysis

HaCaT cells were seeded into 60 mm plates at a density of 1 × 10^6^ cells per well and treated with 0.25 and 0.5 µg/mL LCPE, with or without 1 µg/mL LPS (Sigma-Aldrich, Merck KGaA, Darmstadt, Germany) for 24 at 37 °C in an incubator. Then, using radioimmunoprecipitation assay (RIPA) buffer (iNtRON Biotechnology in Gyeonggi, Korea) that contained a protease inhibitor cocktail and a phosphatase inhibitor (Thermo Fisher Scientific in Waltham, MA, USA), the incubated cells were lysed. Following the manufacturer’s instructions, the bicinchoninic acid (BCA) assay (Thermo Fisher Scientific, Waltham, MA, USA) was used to quantify the protein content of each cell lysate sample. In 10–15% Sodium Dodecyl Sulfate–Polyacrylamide Gel Electrophoresis (SDS PAGE), equal volumes of protein (10 µg) were isolated. The creation of polyacrylamide gels was followed by their transfer to polyvinylidene fluoride (PVDF) membranes (ATTO Co., Ltd., Tokyo, Japan) using a semi-dry transfer system (JP/WSE-4040 HorizeBLOT 4M-R WSE-4045; Atto Corp., Tokyo, Japan). Then, the membranes were blocked with EzBlockChemi (ATTO Blotting System, Tokyo, Japan) for 2 h at room temperature. Membranes were further incubated overnight at 4 °C with 1:1000-diluted primary antibodies. The membranes were washed 5 times for 15 min with Tween 20 (TBS-T, pH7.4) and incubated with 1:5000-diluted anti-rabbit and anti-mouse antibodies (cat. no. A120-101P, Bethyl Laboratory, Inc., Montgomery, TX, USA) for 3 h at room temperature. The membranes were then rewashed using TBS-T 10 times for 2 h. The proteins were detected with enhanced chemiluminescence (ECL) buffer (Bio-Rad, Hercules, CA, USA), and the images were acquired using the ChemiDoc imaging system (Version 6.0, Bio-Rad Laboratories, Inc., Hercules, CA, USA) and analyzed using the Image Lab 4.1 (Bio-Rad) program. The loading control was the β-actin protein, and Western blot images were quantified using the Image J software (https://imagej.nih.gov/ij/download.html, U.S. National Institutes of Health, Bethesda, MD, USA).

### 2.7. Molecular Docking Analysis

Molecular docking analysis was carried out by retrieving the protein structure from PDB (https://www.rcsb.org/, accessed on 10 May 2023) using the search ID 4Q3J (NF-кB), and the 3D compound structures of chlorogenic acid (Compound CID: 1794427), narirutin (Compound CID: 442431), and isorhamnetin (Compound CID: 5281654) were downloaded from PubChem (https://pubchem.ncbi.nlm.nih.gov/, accessed on 10 May 2023). With the default settings, docking analysis was carried out using UCSF Chimera and AutoDock Vina. The docking results were visualized using PyMOL and Discovery Studio (DeLano, 2002). Total intermolecular energy and estimated free energy binding were used to calculate the binding affinities.

### 2.8. Statistical Analysis

The data are expressed as the mean ± SEM. Software called GraphPad Prism (version 9.3.1; GraphPad Software, Inc.) was used to analyze the data. Statistical analysis was performed using SPSS version 12.0 (SPSS Inc., Chicago, IL, USA). One-way factorial analysis of variance (ANOVA) was used to determine whether there were significant differences between the groups. Dunnett’s multiple-comparison tests were then conducted, and *p* <0.05 was regarded as statistically significant (# *p* < 0.05, ## *p* < 0.01, ### *p* < 0.001 vs. untreated, positive control group; * *p* < 0.05, ** *p* < 0.01, *** *p* < 0.001 vs. LPS-treated, negative control group).

## 3. Results and Discussion

### 3.1. Separation and Characterization of Polyphenols in LCPE

**Figure 1:**
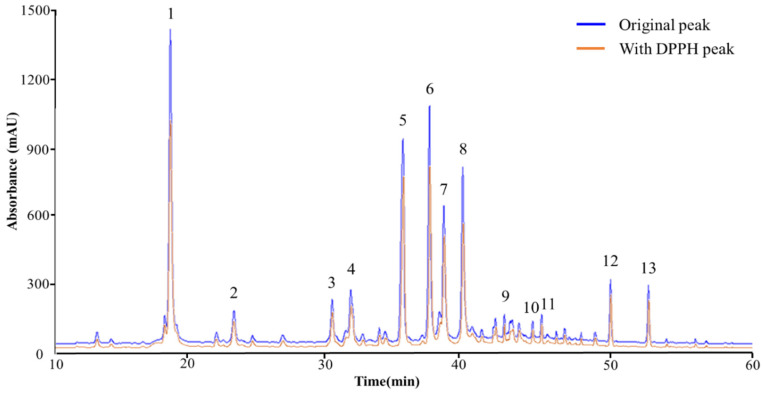
The HPLC chromatograms of the phenolic compounds in LCPE. While the orange line represents the chromatogram following the reaction with the DPPH solution, the blue line represents the initial chromatogram of LCPE before the reaction. The compounds detected at the 284 nm wavelength are chlorogenic acid (1), sweroside (2), isoliquiritin (3), liquiritin (4), narirutin (5), isochlorogenic acid (6), quercitrin (7), isorhamnetin (8), isoliquiritigenin (9), arctiin (10), kaempferol-3-O-rutinoside (11), hesperidin (12), and arctigenin (13).

HPLC-MS/MS was used to perform both quantitative and qualitative analyses of the compounds found in LCPE. A total of 13 peaks were obtained from the HPLC retention times and UV-vis spectra (Figure 1). HPLC chromatography was used to identify the peaks of 13 phenolic compounds at a wavelength of 248 nm. The 13 polyphenol compounds were chlorogenic acid [17], sweroside [18], isoliquiritin [19], liquiritin [20], narirutin [21], isochlorogenic acid A [22], quercitrin [23], isorhamnetin [24], isoliquiritigenin [25], arctiin [26], kaempferol-3-O-rutinoside [27], hesperidin [28], and arctigenin [29]. The results are based on fragmentation patterns. The 13 polyphenolic compounds were quantified using mass spectrometry data from published sources, as shown in Table 1.

**Figure 2:**
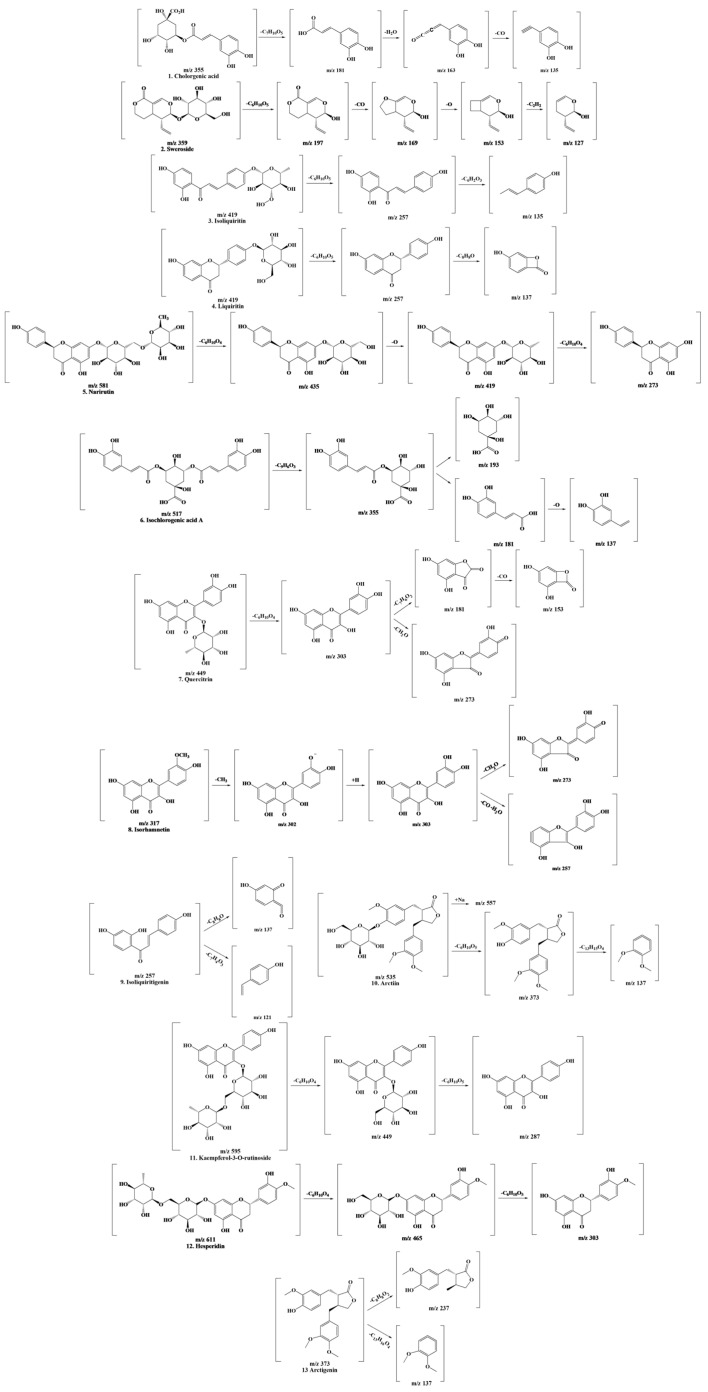
Fragmentation scheme of the polyphenols contained in LCPE.

The following physiologically active compounds are results that can differ depending on the growth conditions or the environment of the plant. However, this study focused on characterizing the detected polyphenolic compounds. Polyphenols were identified based on peaks and mass patterns of molecular ions, as determined by LC-MS/MS and comparison with previously reported literature data [18,19,20,21,22,23,24,25,26,27,28,29,30]. The outcomes of predicting the cleavage of compounds using LC-MS/MS data are shown in Figure 2.

### 3.2. Screening of Antioxidant Polyphenolic Compounds in LCPE

The search for potential antioxidant candidates present in LCPE was conducted using DPPH-HPLC analysis. In general, the DPPH-radical-scavenging activity assay is used when examining the antioxidant effect, which is useful for confirming the antioxidant activity of complex compounds contained in natural products. The violet DPPH (2,2-Diphenyl-1-picrylhdrazyl) reagent contains free radicals, which are removed by the hydrogen atom of the antioxidant compound, and the electrons of the antioxidant provide electrons to free radicals. The solution turns yellow due to the combination of the antioxidant and DPPH reagent due to the mixing mechanism. At this time, the lower the binding force between the hydrogen atom and the antioxidant and the lower the ionization energy of the electrons of the antioxidant, the easier the electrons are transferred. These two are important parameters for measuring antioxidant power [30]. According to the results of HPLC-MS/MS obtained after DPPH bound to and reacted with LCPE, as shown in Figure 1, LCPE contains various bioactive substances that change. In Table 2, the change in the peak area value represents the competitive reaction of the compound with DPPH. In addition, in the reaction with DPPH, the difference in peak area values before and after DPPH binding indicates higher radical-scavenging activity.

In Table 2, the antioxidant effect can be confirmed through the difference between the initial peak area value of each polyphenol compound in LCPE and the area value after the DPPH reaction. Chlorogenic acid showed the largest reaction area with 5255.67 mAU, but isorhamnetin had the reaction area value with the highest change rate of 29.47%. Chlorogenic acid and narirutin followed with 26.82 and 23.9%, respectively. First of all, chlorogenic acid is a plant polyphenol present in coffee, tea, and herbs and is known to have anti-inflammatory effects by relieving oxidative stress and regulating a number of important metabolic pathways [31]. Narirutin is also a flavanone, a natural phytochemical that is abundant in citrus peels and has anticancer, neuroprotective, antioxidant, and anti-inflammatory effects [32]. Isorhamnetin is also known to have anti-inflammatory and antioxidant effects through the regulation of various pathways [33].

These results suggest that three compounds (chlorogenic acid, narirutin, and isorhamnetin) account for the main antioxidant activity of LCPE. Among them, isorhamnetin, chlorogenic acid, and narirutin, which have high area ratios after the reaction, appear to have more effective antioxidant activity.

### 3.3. Anti-Inflammatory Effects of LCPE

Free radicals caused by various biological and environmental factors cause inflammatory diseases and increase the production of ROS in damaged areas due to increased oxygen absorption by leukocytes and mast cells in the inflammatory response [10]. Based on the antioxidant effect of this LCPE, we confirmed an additional in vitro anti-inflammatory effect. To identify various anti-inflammatory factors of LCPE for dermatitis, a model in which inflammation was induced by treating skin keratinocytes with LPS was used. Allergic and atopic skin diseases appear as an inflammatory response due to an excessive immune response. Since keratinocytes are closely related to the immune response, the anti-inflammatory effect of LCPE was confirmed using a model in which inflammation was induced by LPS in HaCaT cells [34].

#### 3.3.1. Effects of LCPE on HaCaT Cell Viability

**Figure 3:**
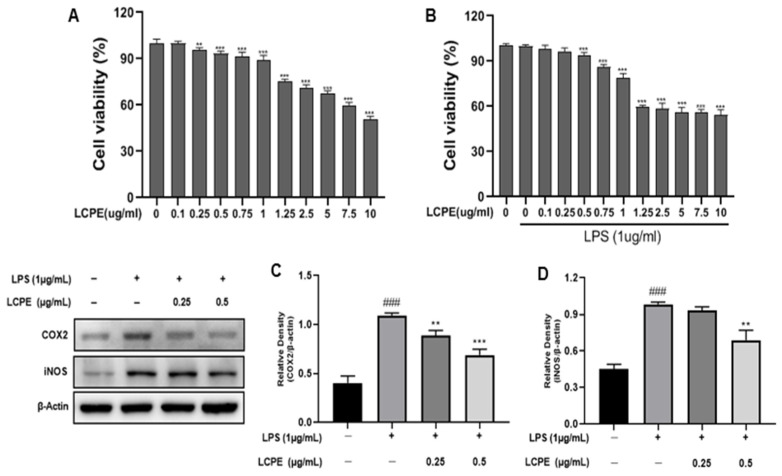
Cytotoxic effect of LCPE on HaCaT cells and the inhibition of inflammatory cytokine proteins by LCPE in HaCaT cells with LPS-induced inflammation. HaCaT cells were pretreated without or with LPS (1 µg/mL) for 1 h at 37 °C. Then, cells were treated with LCPE (0, 0.1, 0.25, 0.5, 0.75, 1, 1.25, 2.5 5, 7.5, 10 µg/mL) for 24 h at 37 °C. (**A**) Cytotoxic effect of LCPE on non-LPS-induced HaCaT cells. (**B**) Cytotoxic effect of LCPE on LPS-induced cell viability in HaCaT cells. HaCaT cells were treated with LCPE (0, 0.25, and 0.5 µg/mL) at indicated concentrations for 24 h. COX2 and iNOS levels were quantified. (**C**) The relative density of COX2 and (**D**) the relative density of iNOS. In comparison to the control group, the findings from three independent experiments are expressed as the mean and standard error of the mean (SEM). ^###^ *p* < 0.001 vs. untreated group; ** *p* < 0.01, *** *p* < 0.001 vs. LPS-treated group.

The 3-(3,4-dimethyl-thiazolyl-2)-2,5-diphenyl tetrazolium bromide (MTT) assay was conducted on HaCaT keratinocytes to assess the cytotoxicity of the extract (Figure 3A,B). LCPE was used to treat HaCaT cells at concentrations of 0, 0.1, 0.25, 0.5, 0.75, 1, 1.25, 2.5, 5, 7.5, and 10 µg/mL for 24 h with or without 1 µg/mL LPS. Figure 3A demonstrates the non-toxicity of the extract at concentrations of 0.25 and 0.5 µg/mL. Therefore, those doses were used in subsequent experiments, as they were not thought to be cytotoxic to HaCaT cells.

#### 3.3.2. Inhibition of COX2 and iNOS Expression in LPS-Induced HaCaT Cells by LCPE

LPS releases various inflammatory cytokines through the activation of MAPK and NF-кB. Among them, iNOS is a representative of pro-inflammatory enzymes that produce nitric oxide (NO) [11,35]. COX2, when stimulated by LPS, acts as an inducer of arachidonic acid conversion and is an enzyme that produces prostaglandins that cause inflammation. It is transcribed through the MAPK or NF-кB pathway due to inflammatory stimulation [36,37].

**Figure 4:**
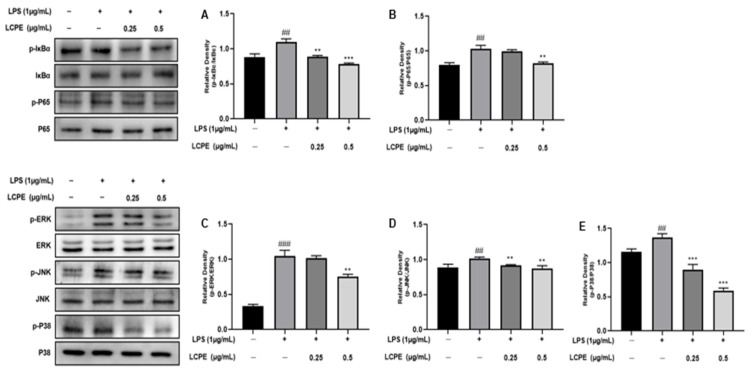
The inhibition of NF-кB and MAPK pathways by LCPE in HaCaT cells with LPS-induced inflammation. The HaCaT cells were treated with LCPE (0, 0.25, and 0.5 µg/mL) at indicated concentrations for 24 h, and p-IкBα and p-P65 levels were quantified. (**A**) The relative density of p-IкBα and (**B**) the relative density of p-P65. The HaCaT cells were treated with LCPE (0, 0.25, and 0.5 µg/mL) at indicated concentrations for 24 h, and p-ERK, p-JNK, and p-P38 levels were quantified. (**C**) The relative density of p-ERK, (**D**) the relative density of p-JNK, and (**E**) the relative density of p-P38. In comparison to the control group, the findings from three independent experiments are expressed as the mean and standard error of the mean (SEM). ^##^ *p* < 0.01, ^###^ *p* < 0.001 vs. untreated group; ** *p* < 0.01, *** *p* < 0.001 vs. LPS-treated group.

Western blotting was employed to examine the impact of LCPE on the expression of two pro-inflammatory cytokines (COX2 and iNOS) on HaCaT cells (Figure 3C,D). Additionally, Figure 4 shows that HaCaT cells that had been exposed to LPS expressed COX-2 at much higher levels. The expression of COX-2 was, however, markedly downregulated in LPS-induced HaCaT cells after LCPE treatment at concentrations of 0.25 and 0.5 µg/mL. These findings indicate that LCPE suppresses COX-2 expression in LPS-induced groups.

#### 3.3.3. Inhibition of NF-кB Pathway in LPS-Induced HaCaT Cells by LCPE

NF-кB is a family of transcription factors that play pivotal roles in regulating genes involved in various processes in the immune and inflammatory responses [38]. In the inflammatory response, NF-кB plays a role in regulating the activation of inflammatory T cells or differentiation; NF-кB activation induces chronic inflammation, and regulating this pathway is a key part of the treatment strategy for inflammatory diseases [39].

Also, LCPE’s effect on NF-кB (IкBα and P65) expression in HaCaT cells was investigated by Western blot analysis (Figure 4A,B). The phosphorylation of IкBα was significantly reduced in LPS-induced HaCaT cells after LCPE treatment at concentrations of 0.25 and 0.5 µg/mL. Also, the phosphorylation of P65 was reduced at a high concentration. It can be seen that inflammation is inhibited through the NF-кB pathway as a result of LCPE treatment in HaCaT cells in which inflammation was induced by LPS.

#### 3.3.4. Inhibition of MAPK Pathway in LPS-Induced HaCaT Cells by LCPE

The mitogen-activated protein kinase (MAPK) pathway is involved in proliferation, differentiation, and cell survival and also plays an important role in the inflammatory response, causing the release of inflammatory cytokines due to the increased expression of target genes through transcription factors such as AP-1 [40]. Extracellular-signal-regulated kinase 1/2 (ERK1/2), c-Jun NH-2-terminal kinase (JNK), and p38-MAPK are important proteins involved in the MAPK pathway [41].

The expression of phosphorylated proteins (ERK1/2, JNK, P38) related to the MAPK pathway was observed through Western blot analysis by applying LCPE treatment at 0.25 and 0.5 µg/mL to HaCaT cells inflamed with LPS (Figure 4C–E). The phosphorylation of ERK1/2 was inhibited in LPS-induced HaCaT cells after LCPE treatment at a high concentration of 0.5 µg/mL. In addition, the phosphorylation of p38 was significantly reduced at concentrations of 0.25 and 0.5 µg/mL. The phosphorylation of JNK decreased in concentration, but it was not significant. In the MAPK pathway, ERK1/2 responds to inflammatory stimuli, but JNK and p38 are activated in response to inflammatory stimuli and the cellular stress environment [42]. Of course, the downregulation of phosphorylated ERK1/2, JNK, and p38 was confirmed, but a significant decrease in relative p-p38 indicates an inflammatory effect, likely due to environmental stress, growth factors, and cytokines, as well as inflammatory stimuli.

**Figure 5:**
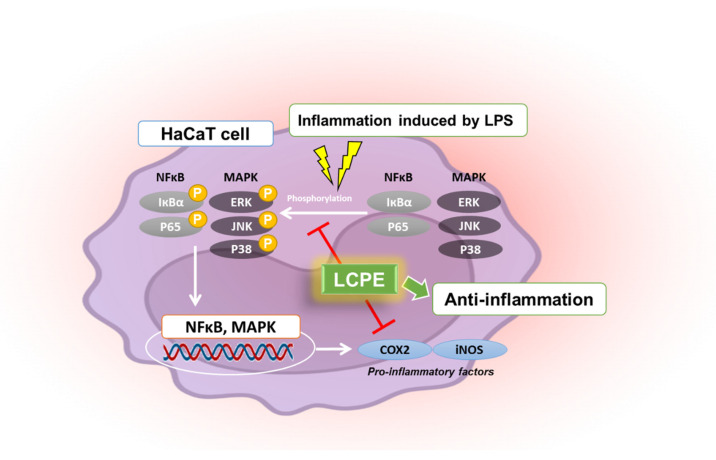
Schematic illustration of anti-inflammatory effects of LCPE in HaCaT cells. This diagram was created by BioRender.

Following antioxidant experiments, additional anti-inflammatory experiments confirmed that the phosphorylation of COX2 and iNOS, which are representative inflammatory factors, was suppressed, and the phosphorylation of MAPK- and NF-кB-pathway-related factors involved in inflammation was also suppressed in inflammation-induced keratinocytes (Figure 5).

### 3.4. Molecular Docking of Chlorogenic Acid, Narirutin, Isorhamnetin, and Isoliquiritigenin with NF-кB

Chlorogenic acid, narirutin, and isorhamnetin are candidate polyphenolic compounds that are considered to have high peak-area change ratios and to have antioxidant effects based on the results of DPPH-binding HPLC (Table 2). Isoliquiritigenin, which had the lowest peak-area change rate, as reported in Table 2, and three compounds (chlorogenic acid, narirutin, and isorhamnetin) were compared and analyzed through molecular docking with NF-кB, a representative anti-inflammatory factor.

**Figure 6:**
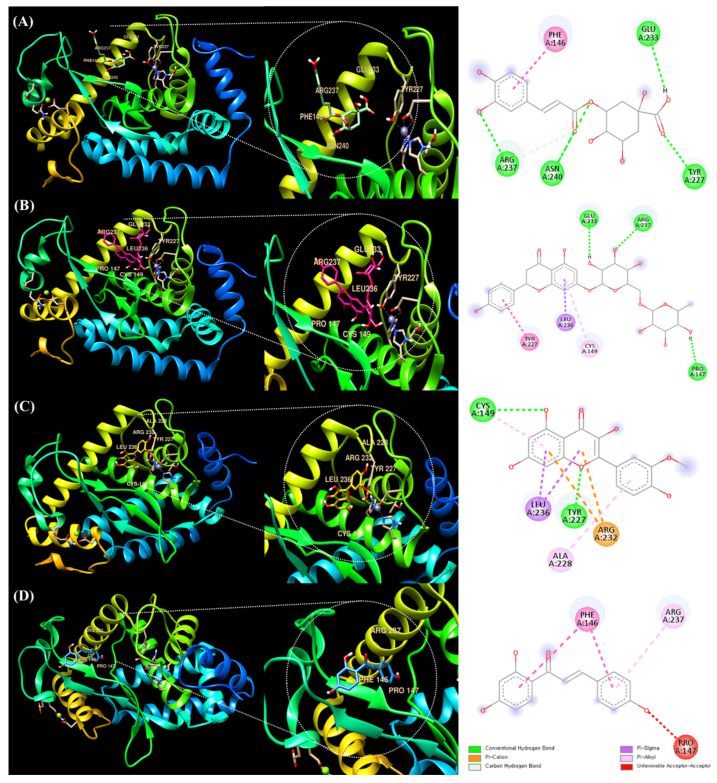
Molecular docking analysis of NF-кB and polyphenolic compounds in LCPE. The 3D structure of NF-кB bound efficiently to (**A**) chlorogenic acid, (**B**) narirutin, (**C**) isorhamnetin, and (**D**) isoliquiritigenin.

The UCSF Chimera program was used to analyze ligand–protein docking. Figure 6A shows that the active site is occupied by chlorogenic acid and NF-кB. It has also been demonstrated that several active sites facilitate ligand binding. The following molecules were found to be active sites in the binding of NF-кB to chlorogenic acid: ARG237, ASN240, GLU233, PHE146, and TYR227 (Table 3). It was discovered that the molecular binding energy score was −6.6 kcal/mol.

Figure 6B shows that the active site is occupied by narirutin and NF-кB. It has been proven that a number of active sites promote ligand binding. The binding of NF-кB to narirutin was discovered to involve the following molecules as active sites: ARG237, CYS149, GLU233, LEU236, PRO147, and TYR227 (Table 3). The docking result for narirutin showed higher binding strength with the binding site than chlorogenic acid, and the molecular binding energy score was −7.4 kcal/mol.

Isorhamnetin and NF-кB are present at the active site, as shown in Figure 6C. Additionally, it has been proven that a number of active sites promote ligand binding. The following molecules were found to be active sites in the binding of NF-кB to isorhamnetin: ARG232, ALA228, CYS149, TYR227, and LEU236 (Table 3). The docking result for isorhamnetin was similar to that of narirutin, but the molecular binding energy score was −7.5 kcal/mol, the highest.

As seen in Figure 6D, the active site contains isoliquiritigenin and NF-кB. It has also been demonstrated that many active sites promote ligand binding. The binding of isoliquiritigenin to NF-B was discovered to involve the following molecules as active sites: ARG237, PHE146, and PRO147 (Table 3). The molecular binding energy score was −6.0 kcal/mol. It was lower than those of the other three polyphenolic compounds (chlorogenic acid, narirutin, and isorhamnetin).

The docking scores of three polyphenolic compounds (chlorogenic acid, narirutin, and isorhamnetin) were higher than that of isoliquiritigenin. This suggests that, in addition to their antioxidant effects, these three compounds would be sufficient anti-inflammatory candidates in terms of anti-inflammatory-related molecular structure binding.

As mentioned earlier, it is important to keep in mind that the outcomes of the molecular docking procedure are not evaluated by determining the precise binding affinity. Here, the absolute docking result value is meaningless, and comparing the outcome values between structures is the key purpose. Additionally, even when a high value is attained, there are instances where the binding structure does not truly bind, so the molecular docking result unavoidably calls for visually verifying structural binding [15].

Based on these results, docking scores were obtained for the binding of the polyphenol compounds in LCPE that had the most significant effect on anti-inflammatory-related proteins. These docking results suggest that LCPE, containing a variety of herbal extracts, has strong antioxidant and also anti-inflammatory effects. Furthermore, we suggest that it can be an effective drug for dermatitis according to the predicted docking data, as well as in vitro results.

## 4. Conclusions

LCPE is a complex extract in which various medicinal materials are mixed. In the complex extract called LCPE, polyphenol compounds were confirmed, and antioxidant and anti-inflammatory effects were observed. Clearly, each compound has antioxidant and anti-inflammatory effects, but synergistic effects of the various polyphenolic compounds are also to be expected.

In this study, the polyphenol compounds in LCPE were identified, and through a combined analysis based on DPPH and HPLC, compounds with the potential to have high antioxidant activity were identified. In addition, through the molecular docking of representative inflammation-related receptors, NF-кB, and the compounds, it was confirmed that the components of LCPE had a significantly high binding score in terms of structural binding.

Therefore, the composition of polyphenolic compounds in these complex extracts, their antioxidant effects, their anti-inflammatory effects on skin inflammation, and their molecular structural binding suggest that LCPE is a potential drug for various inflammation-related pathways, with structural affinity and antioxidant effects. The results also suggest that it could be a promising and predictable drug model for treating purulent dermatitis and reducing inflammation.

## Figures and Tables

**Table 1:** The HPLC-MS/MS data of phenolic compounds from LCPE.

Peak No.	Rt (Min)	Formula	Compound	UV Max	[M + H]^+^	MS/MS
1	18.149	C_16_H_18_O_9_	Chlorogenic acid	325, 250	355	181 (C_9_H_8_O_4_) [M + H-C_7_H_10_O_5_]^+^ 163 (C_9_H_6_O_3_) [M + H-C_7_H_10_O_5_-H_2_O]^+^ 135 (C_8_H_6_O_2_) [M + H-C_7_H_12_O_6_-CO]^+^
2	22.719	C_16_H_22_O_9_	Sweroside	245	359	197 (C_10_H_12_O_4_) [M + H-C_6_H_10_O_5_]^+^ 169 (C_9_H_12_O_3_) [M + H-C_6_H_10_O_5_-CO]^+^ 153 (C_9_H_12_O_2_) [M + H-C_7_H_10_O_6_-O]^+^ 127 (C_7_H_10_O_2_) [M + H-C_7_H_10_O_7_-C_2_H_2_]^+^
3	29.784	C_21_H_22_O_9_	Isoliquiritin	365, 235	419	257 (C_15_H_12_O_4_) [M + H-C_6_H_10_O_5_]^+^
4	31.097	C_21_H_22_O_9_	Liquiritin	275, 235	419	257 (C_15_H_12_O_4_) [M + H-C_6_H_10_O_5_]^+^ 137 (C_7_H_4_O_3_) [M + H-C_6_H_10_O_5_-C_8_H_8_O]^+^
5	34.840	C_27_H_32_O_14_	Narirutin	285, 220	581	435 (C_21_H_22_O_10_) [M + H-C_6_H_10_O_4_]^+^ 419 (C_21_H_22_O_10_) [M + H-C_6_H_10_O_4_-O]^+^ 273 (C_15_H_12_O_5_) [M + H-C_6_H_10_O_5_-C_6_H_10_O_4_]^+^
6	36.749	C_25_H_24_O_12_	Isochlorogenic acid A	330, 290	517	355 (C_16_H_18_O_9_) [M + H-C_9_H_6_O_3_]^+^ 193 (C_7_H_12_O_6_) [M + H-C_9_H_6_O_3_-C_9_H_6_O_3_]^+^ 181 (C_9_H_8_O_4_) [M + H-C_9_H_6_O_3_-C_7_H_10_O_5_]^+^ 137 (C_8_H_8_O_2_) [M + H-C_16_H_16_O_8_-O]^+^
7	37.813	C_21_H_20_O_11_	Quercitrin	350, 260	449	303 (C_15_H_10_O_7_) [M + H-C_6_H_10_O_4_]^+^ 273 (C_14_H_8_O_6_) [M + H-C_6_H_10_O_4_-CH_2_O]^+^ 181 (C_8_H_4_O_5_) [M + H-C_6_H_10_O_4_-C_7_H_6_O_2_]^+^ 153 (C_7_H_4_O_4_) [M + H-C_13_H_16_O_6_-CO]^+^
8	39.147	C_16_H_12_O_7_	Isorhamnetin	350, 255	317	302 (C_15_H_9_O_7_^-^) [M + H-CH_3_]^+^ 303 (C_15_H_10_O_7_) [M + H-CH_3_ + H]^+^ 273 (C_14_H_8_O_6_) [M + H-CH_2_-CH_2_O]^+^ 257 (C_14_H_8_O_5_) [M + H-CH_2_-CO-H_2_O]^+^
9	42.124	C_15_H_12_O_4_	Isoliquiritigenin	370, 240	257	137 (C_7_H_4_O_3_) [M + H-C_8_H_8_O]^+^ 121 (C_8_H_8_O) [M + H-C_7_H_4_O_3_]^+^
10	44.192	C_27_H_34_O_11_	Arctiin	278, 225	535	557 (C_27_H_34_O_11_Na) [M + H + Na]^+^ 373 (C_21_H_24_O_6_) [M + H-C_6_H_10_O_5_]^+^ 137 (C_8_H_9_O_2_^+^) [M + H-C_6_H_10_O_5_-C_12_H_15_O_4_]^+^
11	44.805	C_27_H_30_O_15_	Kaempferol-3-O-rutinoside	340, 270	595	449 (C_21_H_20_O_11_) [M + H-C_6_H_10_O_4_]^+^ 287 (C_15_H_10_O_6_) [M + H-C_6_H_10_O_4_-C_15_H_10_O_6_]^+^
12	49.781	C_28_H_34_O_15_	Hesperidin	280, 225	611	465 (C_22_H_24_O_11_) [M + H-C_6_H_10_O_4_]^+^ 303 (C_16_H_14_O_6_) [M + H-C_6_H_10_O_4_-C_6_H_10_O_5_]^+^
13	52.497	C_21_H_24_O_6_	Arctigenin	280, 230	373	237 (C_14_H_16_O_4_) [M + H-C_8_H_8_O_2_]^+^ 137 (C_8_H_8_O_2_) [M + H-C_13_H_16_O_4_]^+^

Rt: retention time.

**Table 2:** Screening of antioxidant potential of LCPE compounds.

Peak No.	Compound	Initial Area (mAU)	Area after DPPH Reaction (mAU)	Reactive Area (mAU)/(%)
1	Chlorogenic acid	19,594.67 ± 49.94 ^K^	14,339 ± 7767 ^L^	5255.67 ± 77.66 ^J^ (26.82 ± 0.38 ^D^)
2	Sweroside	2307.67 ± 51.94 ^D^	1852 ± 50.03 ^E^	455.67 ± 40.80 ^C^ (19.74 ± 1.61 ^B^)
3	Isoliquiritin	3051.33 ± 44.38 ^E^	2360 ± 37.04 ^F^	691.33 ± 56.08 ^D^ (22.65 ±1.62 ^C^)
4	Liquiritin	4449.67 ± 37.90 ^F^	3428 ± 54.67 ^G^	1021.67 ±22.01 ^E^ (22.96 ± 0.64 ^C^)
5	Narirutin	15,566.33 ± 16.62 ^J^	11,835.33 ± 36.47 ^K^	3731 ± 22.72 ^I^ (23.97 ± 0.17 ^C^)
6	Isochlorogenic acid A	14,661.67 ± 23.03 ^I^	11,223.67 ±76.23 ^J^	3438 ± 54.15 ^H^ (23.45 ± 0.40 ^C^)
7	Quercitrin	8555.67 ± 76.84 ^G^	6594.67 ± 49.69 ^H^	1961 ± 44.44 ^F^ (22.92 ± 0.38 ^C^)
8	Isorhamnetin	10,751.67 ± 14.57 ^H^	7582.67 ± 25.40 ^I^	3169 ± 37.03 ^G^ (29.47 ± 0.31 ^E^)
9	Isoliquiritigenin	731.33 ± 5.69 ^A^	660.67 ± 6.43 ^B^	70.67 ±3.79 ^A^ (9.66 ± 0.51 ^A^)
10	Arctiin	757.33 ± 28.02 ^A^	587 ± 9.17 ^A^	170.33 ± 19.22 ^B^ (22.45 ± 1.70 ^C^)
11	Kaempferol-3-O-rutinoside	946.33 ± 29.01 ^B^	756.33 ± 25.58 ^C^	190 ± 11 ^B^ (20.08 ± 1.02 ^B^)
12	Hesperidin	2278.67 ± 13.28 ^D^	1855 ± 14.73 ^E^	423.67 ± 27.59 ^C^ (18.59 ± 1.10 ^B^)
13	Arctigenin	2160 ± 16.52 ^C^	1737.33 ± 8.14 ^D^	422.67 ± 8.74 ^C^ (19.57 ± 0.26 ^B^)

All values are mean ± SD (*n* = 3). ^A–L^ Means with different superscripts in the same column are significantly different at *p* < 0.05 by Duncan’s multiple range tests. Also tested in the same column based on superscript ^A^.

**Table 3:** Molecular docking studies of chlorogenic acid, narirutin, isorhamnetin, and isoliquiritigenin with NF-кB complex and their binding energies.

Binding Ligand	Amino Acid Residue That Interacts	Docking Score
Chlorogenic acid	ARG237, ASN240, GLU233, PHE146, TYR227	−6.6 kcal/mol
Narirutin	ARG237, CYS149, GLU233, LEU236, PRO147, TYR227	−7.4 kcal/mol
Isorhamnetin	ARG232, ALA228, CYS149, TYR227, LEU236	−7.5 kcal/mol
Isoliquiritigenin	ARG237, PHE146, PRO147	−6.0 kcal/mol

## Data Availability

The data used to support the findings of this study are available upon request from the corresponding author.

## References

[1] Schilrreff P., Alexiev U. (2022). Chronic Inflammation in Non-Healing Skin Wounds and Promising Natural Bioactive Compounds Treatment. Int. J. Mol. Sci..

[2] Chan C.X., Zug K.A. (2021). Diagnosis and Management of Dermatitis, Including Atopic, Contact, and Hand Eczemas. Med. Clin. N. Am..

[3] Clark G.W., Pope S.M., Jaboori K.A. (2015). Diagnosis and treatment of seborrheic dermatitis. Am. Fam. Physician.

[4] Li Y., Li W., Fu C., Song Y., Fu Q. (2020). Lonicerae japonicae flos and Lonicerae flos: A systematic review of ethnopharmacology, phytochemistry and pharmacology. Phytochem. Rev..

[5] Maurya A.K., Mohanty S., Pal A., Chanotiya C.S., Bawankule D.U. (2018). The essential oil from Citrus limetta Risso peels alleviates skin inflammation: In-vitro and in-vivo study. J. Ethnopharmacol..

[6] Yin L., Guan E., Zhang Y., Shu Z., Wang B., Wu X., Chen J., Liu J., Fu X., Sun W. (2018). Chemical Profile and Anti-inflammatory Activity of Total Flavonoids from Glycyrrhiza Uralensis Fisch. Iran J. Pharm. Res..

[7] Chen G.L., Fan M.X., Wu J.L., Li N., Guo M.Q. (2019). Antioxidant and anti-inflammatory properties of flavonoids from lotus plumule. Food Chem..

[8] Nile S.H., Keum Y.S., Nile A.S., Jalde S.S., Patel R.V. (2018). Antioxidant, anti-inflammatory, and enzyme inhibitory activity of natural plant flavonoids and their synthesized derivatives. J. Biochem. Mol. Toxicol..

[9] Pan M.H., Chiou Y.S., Tsai M.L., Ho C.T. (2011). Anti-inflammatory activity of traditional Chinese medicinal herbs. J. Tradit. Complement. Med..

[10] Arulselvan P., Fard M.T., Tan W.S., Gothai S., Fakurazi S., Norhaizan M.E., Kumar S.S. (2016). Role of Antioxidants and Natural Products in Inflammation. Oxid. Med. Cell. Longev..

[11] Kim S.-Y., Hong M., Deepa P., Sowndhararajan K., Park S.J., Park S., Kim S. (2023). Carthamus tinctorius Suppresses LPS-Induced Anti-Inflammatory Responses by Inhibiting the MAPKs/NF-&kappa;B Signaling Pathway in HaCaT Cells. Sci. Pharm..

[12] Lin C.Y., Wang W.H., Chen S.H., Chang Y.W., Hung L.C., Chen C.Y., Chen Y.H. (2017). Lipopolysaccharide-Induced Nitric Oxide, Prostaglandin E2, and Cytokine Production of Mouse and Human Macrophages Are Suppressed by Pheophytin-b. Int. J. Mol. Sci..

[13] Liou C.J., Len W.B., Wu S.J., Lin C.F., Wu X.L., Huang W.C. (2014). Casticin inhibits COX-2 and iNOS expression via suppression of NF-kappaB and MAPK signaling in lipopolysaccharide-stimulated mouse macrophages. J. Ethnopharmacol..

[14] Singh A.N., Baruah M.M., Sharma N. (2017). Structure Based docking studies towards exploring potential anti-androgen activity of selected phytochemicals against Prostate Cancer. Sci. Rep..

[15] Meng X.Y., Zhang H.X., Mezei M., Cui M. (2011). Molecular docking: A powerful approach for structure-based drug discovery. Curr. Comput. Aided Drug Des..

[16] Kim S.M., Lee S.J., Venkatarame Gowda Saralamma V., Ha S.E., Vetrivel P., Desta K.T., Choi J.Y., Lee W.S., Shin S.C., Kim G.S. (2019). Polyphenol mixture of a native Korean variety of Artemisia argyi H. (Seomae mugwort) and its anti-inflammatory effects. Int. J. Mol. Med..

[17] Willems J.L., Khamis M.M., Mohammed Saeid W., Purves R.W., Katselis G., Low N.H., El-Aneed A. (2016). Analysis of a series of chlorogenic acid isomers using differential ion mobility and tandem mass spectrometry. Anal. Chim. Acta.

[18] Han H., Zeng W., He C., Bligh S.W., Liu Q., Yang L., Wang Z. (2014). Characterization of metabolites of sweroside in rat urine using ultra-high-performance liquid chromatography combined with electrospray ionization quadrupole time-of-flight tandem mass spectrometry and NMR spectroscopy. J. Mass. Spectrom..

[19] Guo Y., Shao S., Zhang W., Li C., Meng Z., Sun S., Yang D., Lu S. (2022). Content Determination and Release Characteristics of Six Components in the Different Phases of "Glycyrrhizaglabra-Nux vomica" Decoction by UPLC-MS/MS. Molecules.

[20] Wang P., Wang B., Xu J., Sun J., Yan Q., Ji B., Zhao Y., Yu Z. (2015). Detection and chemical profiling of Ling-Gui-Zhu-Gan decoction by ultra performance liquid chromatography-hybrid linear ion trap-Orbitrap mass spectrometry. J. Chromatogr. Sci..

[21] Wang C., Pan Y., Fan G., Chai Y., Wu Y. (2010). Application of an efficient strategy based on MAE, HPLC-DAD-MS/MS and HSCCC for the rapid extraction, identification, separation and purification of flavonoids from Fructus Aurantii Immaturus. Biomed. Chromatogr..

[22] Peres R.G., Tonin F.G., Tavares M.F., Rodriguez-Amaya D.B. (2013). HPLC-DAD-ESI/MS identification and quantification of phenolic compounds in Ilex paraguariensis beverages and on-line evaluation of individual antioxidant activity. Molecules.

[23] Li A., Hou X., Wei Y. (2018). Fast screening of flavonoids from switchgrass and Mikania micrantha by liquid chromatography hybrid-ion trap time-of-flight mass spectrometry. Anal. Methods.

[24] Raza S., Chaudhary A., Mumtaz M., Adnan A., Mukhtar H., Akhtar M. (2020). Metabolite profiling and antidiabetic attributes of ultrasonicated leaf extracts of *Conocarpus lancifolius*. Asian Pac. J. Trop. Biomed..

[25] Zhao X., Zhang S., Liu D., Yang M., Wei J. (2020). Analysis of Flavonoids in Dalbergia odorifera by Ultra-Performance Liquid Chromatography with Tandem Mass Spectrometry. Molecules.

[26] Liu J., Cai Y.Z., Wong R.N., Lee C.K., Tang S.C., Sze S.C., Tong Y., Zhang Y. (2012). Comparative analysis of caffeoylquinic acids and lignans in roots and seeds among various burdock (*Arctium lappa*) genotypes with high antioxidant activity. J. Agric. Food Chem..

[27] Liu P., Kallio H., Yang B. (2014). Flavonol glycosides and other phenolic compounds in buds and leaves of different varieties of black currant (*Ribes nigrum* L.) and changes during growing season. Food Chem..

[28] Bhatt V., Sharma S., Kumar N., Sharma U., Singh B. (2017). Simultaneous quantification and identification of flavonoids, lignans, coumarin and amides in leaves of Zanthoxylum armatum using UPLC-DAD-ESI-QTOF-MS/MS. J. Pharm. Biomed. Anal..

[29] Zou Q., Gu Y., Lu R., Zhang T., Zhao G.R., Liu C., Si D. (2013). Development of an LC/MS/MS method in order to determine arctigenin in rat plasma: Its application to a pharmacokinetic study. Biomed. Chromatogr..

[30] Kedare S.B., Singh R.P. (2011). Genesis and development of DPPH method of antioxidant assay. J. Food Sci. Technol..

[31] Liang N., Kitts D.D. (2015). Role of Chlorogenic Acids in Controlling Oxidative and Inflammatory Stress Conditions. Nutrients.

[32] Mitra S., Lami M.S., Uddin T.M., Das R., Islam F., Anjum J., Hossain M.J., Emran T.B. (2022). Prospective multifunctional roles and pharmacological potential of dietary flavonoid narirutin. Biomed. Pharmacother..

[33] Gong G., Guan Y.Y., Zhang Z.L., Rahman K., Wang S.J., Zhou S., Luan X., Zhang H. (2020). Isorhamnetin: A review of pharmacological effects. Biomed. Pharmacother..

[34] Kim S.Y., Han S.D., Kim M., Mony T.J., Lee E.S., Kim K.M., Choi S.H., Hong S.H., Choi J.W., Park S.J. (2021). Mentha arvensis Essential Oil Exerts Anti-Inflammatory in LPS-Stimulated Inflammatory Responses via Inhibition of ERK/NF-kappaB Signaling Pathway and Anti-Atopic Dermatitis-like Effects in 2,4-Dinitrochlorobezene-Induced BALB/c Mice. Antioxidants.

[35] Gilroy D.W., Colville-Nash P.R., Willis D., Chivers J., Paul-Clark M.J., Willoughby D.A. (1999). Inducible cyclooxygenase may have anti-inflammatory properties. Nat. Med..

[36] Wang B., Wu L., Chen J., Dong L., Chen C., Wen Z., Hu J., Fleming I., Wang D.W. (2021). Metabolism pathways of arachidonic acids: Mechanisms and potential therapeutic targets. Signal Transduct. Target. Ther..

[37] Jeong Y.E., Lee M.Y. (2018). Anti-Inflammatory Activity of Populus deltoides Leaf Extract via Modulating NF-kappaB and p38/JNK Pathways. Int. J. Mol. Sci..

[38] Liu T., Zhang L., Joo D., Sun S.C. (2017). NF-kappaB signaling in inflammation. Signal Transduct. Target. Ther..

[39] Lawrence T. (2009). The nuclear factor NF-kappaB pathway in inflammation. Cold Spring Harb. Perspect. Biol..

[40] Wu X., Schauss A.G. (2012). Mitigation of inflammation with foods. J. Agric. Food Chem..

[41] Moens U., Kostenko S., Sveinbjornsson B. (2013). The Role of Mitogen-Activated Protein Kinase-Activated Protein Kinases (MAPKAPKs) in Inflammation. Genes.

[42] Zhang W., Liu H.T. (2002). MAPK signal pathways in the regulation of cell proliferation in mammalian cells. Cell Res..

